# Towards Dependent Race Models for the Stop-Signal Paradigm: The Copula Approach

**DOI:** 10.1007/s42113-023-00184-3

**Published:** 2023-11-06

**Authors:** Hans Colonius, Paria Jahansa, Harry Joe, Adele Diederich

**Affiliations:** 1 Department of Psychology, Carl von Ossietzky Universität Oldenburg, Ammerländer Heerstraße 114-118, Oldenburg, 26129 Germany; 2 Department of Statistics, The University of British Columbia, 2207 Main Mall, Vancouver, V6T 1Z4 BC Canada

**Keywords:** Stop signal paradigm, Race model, Copula, Dependent censoring

## Abstract

The race model for stop signal processing is based on the assumption of context independence between the go and stop process. Recent empirical evidence inconsistent with predictions of the independent race model has been interpreted as a failure of context independence. Here we demonstrate that, keeping context independence while assuming stochastic dependency between go and stop processing, one can also account for the observed violations. Several examples demonstrate how stochastically dependent race models can be derived from copulas, a rapidly developing area of statistics. The non-observability of stop signal processing time is shown to be equivalent to a well known issue in random dependent censoring.

## Introduction

The stop-signal paradigm is a popular tool to study response inhibition. In this setting, participants perform a reaction time (RT) *go task*; typically: press the button when a go signal occurs (simple RT); or, press the left button when an arrow pointing to the left appears, and press right when an arrow pointing to the right appears (choice RT). On a minority of trials, a stop signal (e.g. an acoustic stimulus) appears after a variable stop-signal delay (SSD) instructing the participant to suppress the imminent go response *stop task*. If the delay is short enough, subjects are usually able to follow the stop instruction so that no reaction time is registered. Yet, the covert latency of the stopping process is considered to be an important aspect of the response inhibition mechanism. Thus, the main goal of modeling the task is to obtain information about the non-observable stop-signal reaction time (SSRT) that is often utilized as diagnostic tool of inhibitory control capacities in brain cognitive development (e.g. Casey et al. , 2018) and for clinical subpopulations (substance abuse, overeating, pathological gambling, risk taking (e.g., Verbruggen and Logan , 2008).

In the prevalent model, known as the *race model* (Logan & Cowan, 1984), performance in the stop-signal task is represented as a race between two (stochastically) independent random variables representing the go and stop signal processing times, denoted as $T_{go}$ and $T_{stop}$, respectively. If $T_{go}$ is smaller than $T_{stop}+t_d$ ($t_d$ denoting the value of SSD), then a response is given in spite of the stopping instructions, otherwise no reaction time (RT) is registered.

Without making specific distributional assumptions about the random variables, the race model allows one to estimate the mean and variability of SSRT (for reviews, see Logan , 1994; Matzke et al. , 2018; Verbruggen et al. , 2019; Colonius and Diederich , 2023). Moreover, Matzke and colleagues (Matzke et al., 2013) developed parametric versions of the race model assuming ex-Gaussian distributions for $T_{go}$ and $T_{stop}$ that provide an estimate of the entire distribution of SSRT. Using hierarchical Bayesian estimation methods, they show that this model has the advantage of requiring fewer numbers of observations per subject than traditional non-parametric methods.

Although the race model, in both its parametric and non-parametric versions, is generally considered to provide a valid description of the processes underlying performance in the stop signal paradigm, a number of empirical observations have revealed that systematic deviations from the race model’s predictions do sometimes occur. The first result of this paper is to show that, in principle, such deviations can be explained by an effect of stochastic dependency between the “racers” in the model. Second, drawing on the statistical concept of a copula, we outline a general approach to modeling stochastic dependency between go and stop processing. This presents an alternative, or additional, route to recent endeavors to generalize the race model. This paper is to provide a proof-of-concept rather than a guide to straightforward application. In particular, issues of parameter estimation and model testing are left for future work.

The paper is organized as follows. The next section presents a somewhat formal description of the context-independent race model in a general setting and concludes with an expression for estimating the non-observable stop signal distribution in the non-parametric case. After adding the assumption of stochastic independence in the subsequent section, we briefly discuss a well-known inequality test (here called “race model inequality”) and its empirical status. Then, the section “Towards Race Models with Stochastic Dependency: The Copula Approach” introduces the notion of a copula and presents a principled way to estimate the non-observable stop signal distribution for copula-based race models. The following section “The Copula Version of the Inequality” presents sufficient conditions for the inequality to hold that do not imply stochastic independence. Several examples illustrate the condition and provide parameter settings where the inequality is, or is not, violated. The problem of choosing a particular copula is shown to be equivalent to a non-identifiability issue in actuarial science (dependent random censoring), and we summarize recent relevant results from this area in the section “Choosing a Copula: The Roleof Dependent Random Censoring”. The final section reviews several interactive race models and discusses the role played by context and stochastic independence in stop-signal race modeling. Some proofs and definitions concerning copula theory are relegated to the appendices.

## The Context-Independent Race Model

The random variables introduced above, $T_{go}$ and $T_{stop}$, are defined with respect to the experimental condition where both go and stop signal are presented, referred to here as the context STOP. The go signal triggers realization of random variable $T_{go}$ and the stop signal triggers a realization of random variable $T_{stop}$. The bivariate distribution function

1 $$\begin{aligned} H(s,t)=P[T_{go}\le s, T_{stop}\le t]=F_{go,stop}(s,t), \end{aligned}$$

is defined for all non-negative real numbers *s* and *t*, with corresponding density. Throughout, we will assume continuity of all random variables.

The outcome of the race is determined by “the winner”:

$$\begin{aligned} \min \{T_{go},T_{stop}+t_d\}, \end{aligned}$$

where again $t_d$ ($t_d \ge 0$) denotes the stop signal delay (SSD), that is, the time between presentation of the go signal and the stop signal.

The marginal distributions of *H*(*s*, *t*) are denoted as:

$$\begin{aligned} F_{go}(s)&=\Pr [T_{go}\le s, T_{stop}< \infty ] \text{ and } \\ F_{stop}(t)&=\Pr [T_{go}< \infty , T_{stop}\le t ]. \end{aligned}$$

In context GO, defined by the absence of a stop signal, only processing of the go signal occurs.

The most general race model makes no assumption about dependency between the stop and go processes. Note that in statistical modeling of the task, the two different experimental conditions in the paradigm, GO and STOP, refer to two different sample spaces and are therefore statistically unrelated. Thus, in principle, the distribution of $T_{go}$ in context GO, $F_{go}^{*}(s)$, say, could differ from the marginal distribution $F_{go}(s)$ in context STOP. However, the context-independent race model rules this out by adding the important assumption of *context independence*:^1^

### Definition 1

(Context independence) In context GO, the distribution of go signal processing time $T_{go}$ is assumed to be equal to the marginal distribution of $T_{go}$ in context STOP:

2 $$\begin{aligned} F_{go}^*(s) \equiv F_{go}(s) =\Pr [T_{go}\le s, T_{stop}< \infty ] \end{aligned}$$

for all *s*.

In general, context STOP would also have to be indexed by the specific value of SSD ($t_d$) being applied in a given trial, and the same holds for *H*(*s*, *t*) and $F_{stop}(t)$. However, we will assume that SSD *invariance* holds, meaning that one can drop the index $t_d$ throughout without consequences while keeping it as a given (design) parameter. Moreover, $T_{stop}$ is set equal to zero for $t\le t_d$ with probability one.

Under these conditions, the probability of observing a response to the go signal given a stop signal was presented with SSD $=t_d$ [ms] after the go signal, is defined by the *race assumption*,

3 $$\begin{aligned} p_r(t_d)&=\Pr [T_{go}\le T_{stop}+t_d]. \end{aligned}$$

In addition, according to the model, the probability of observing a response to the go signal no later than time *s*, given the stop signal was presented with a delay $t_d$, is given by the (conditional) distribution function denoted as $F_{sr}(s;t_d)$,

4 $$\begin{aligned} F_{sr}(s;t_d)= & {} \Pr [T_{go}\le s| T_{go}<T_{stop}+t_d] \nonumber \\= & {} \frac{\Pr [T_{go}\le s, \, T_{go}<T_{stop}+t_d] }{\Pr [T_{go}<T_{stop}+t_d]}, \end{aligned}$$

also called *signal-respond* distribution. For $0<s\le t_d$, the numerator of Eq. 4 is:

$$\begin{aligned} \Pr [T_{go}\le s, \, T_{go}<T_{stop}+t_d] = F_{go}(s). \end{aligned}$$

To summarize, data obtainable in the stop-signal paradigm are estimates of probability $p_r(t_d)$, distribution $F_{go}$, and conditional distribution $F_{sr}(s ;t_d)$. The main goal is to estimate the distribution $F_{stop}$, or at least some of its moments, from these data in order to obtain information about the non-observable mechanism of stop signal processing.

Let $f_{go}=F'_{go}$ be the density and $F_{stop|go}(\cdot |s)$ the conditional distribution of $[T_{stop}|T_{go}=s]$ for $s>0$. Then, for $s>t_d>0$,

5 $$\begin{aligned}&\Pr [T_{go}\le s, \, T_{go}<T_{stop}+t_d] \nonumber \\&= \int _0^s [1-F_{stop|go}(s'-t_d|s')] \, f_{go}(s')\, \textrm{d}s' \nonumber \\&= \int _0^{t_d} f_{go}(s')\, \textrm{d}s' +\!\! \int _{t_d}^s \!\!\![1-F_{stop|go}(s'-t_d|s')] \, f_{go}(s')\, \textrm{d}s' \nonumber \\&= F_{go}(t_d) + \int _{t_d}^s\!\! [1-F_{stop|go}(s'-t_d|s')] \, f_{go}(s')\, \textrm{d}s' . \end{aligned}$$

From Eqs. 4 and 5,

6 $$\begin{aligned} F_{sr}(s ;t_d)\; p_r(t_d)&= F_{go}(t_d) \nonumber \\&+ \int _{t_d}^s [1-F_{stop|go}(s'-t_d|s')] \, f_{go}(s')\, \textrm{d}s'. \end{aligned}$$

Because $p_r(t_d)$ and the first integral on the right do not depend on *s*, taking the first derivative with respect to *s* yields:

$$\begin{aligned} f_{sr}(s ;t_d)\; p_r(t_d)&=[1-F_{stop|go}(s-t_d|s)] \,f_{go}(s). \end{aligned}$$

We solve for the (conditional) stop signal distribution:

7 $$\begin{aligned} F_{stop|go}(s-t_d|s)= 1-\frac{f_{sr}(s ;t_d)}{f_{go}(s)}\,p_r(t_d), \end{aligned}$$

where all expressions on the right-hand side are “observable”, that is, they can be estimated from data collected in the stop signal task.

### The Independent Race (IND) Model

The dominant version of the race model, the independent race model (IND model, for short), adds the assumption of stochastic independence between $T_{go}$ and $T_{stop}$ to the context-independent model, that is,

8 $$\begin{aligned} H(s,t)= F_{go,stop}(s,t)=F_{go}(s)\cdot \,F_{stop}(t), \end{aligned}$$

for all *s* and *t*.

In this model, mean RT of stop failures, i.e. when a response is given although a stop signal was presented, should not be larger than the mean RT of go responses without a stop signal occurring:

9 $$\begin{aligned} \text{ E }[T_{go}\,|\, T_{go}< T_{stop}+t_d]&\le \text{ E }[T_{go}] , \end{aligned}$$

with $t_d\ge 0$. Moreover, the left-hand side, mean signal-respond RT, should be monotonically increasing with stop signal delay $t_d$. The inequality on the means follows directly from an ordering of the RT distribution functions:

10 $$\begin{aligned} F_{go}(s) \le F_{sr}(s;t_d), \end{aligned}$$

holding for all $s, s>t_d,$ (for a proof, see Colonius et al. 2001).

### The Empirical Status of Inequality $F_{go}(s) \le F_{sr}(s\,;\,t_d)$

In earlier work, we had found some empirical violations of this distribution ordering at short SSDs (e.g., Colonius et al. , 2001; Özyurt et al. 2003) but evidence remained weak because, typically, observations are sparse at short SSDs. Recently, in a large-scale survey analyzing 14 experimental studies, Bissett et al. (2021) observed serious violations of the predicted mean ordering, again mostly at short SSDs (less than 200 ms). Bissett and colleagues interpret their findings as refuting context independence and discuss a number of possible alternative models accommodating context dependence as a function of SSD.

We do not take a stance on how sweeping the empirical evidence against context independence is given these findings. Developing explicit models incorporating context dependency seems a worthwhile enterprise in any case (see “Discussion” section).

Alternatively, here we want to explore whether the observed violations can be accommodated by dropping the assumption of stochastic independence between go and stop signal processing while keeping context independence. This is prompted by the finding, detailed below, that there exist context-independent race models that nonetheless violate inequalities (9) and (10) when $T_{go}$ and $T_{stop}$ are assumed to be stochastically *dependent* random variables with specific distributions. Thus, violation of the inequalities may indicate that either context or stochastic independence, or both, may fail but it seems difficult to tell them apart without additional information.

## Towards Race Models with Stochastic Dependency: The Copula Approach

Let us assume that context independence holds; Inequality (10) equals

11 $$\begin{aligned} \Pr [T_{go}\le t]\le \Pr [T_{go}\le t\,|\, T_{go}< T_{stop}+t_d]. \end{aligned}$$

As mentioned above, inequality (11) holds for stochastically independent $T_{go}$ and $T_{stop}$ (Colonius et al., 2001). However, pinning down the type of stochastic dependency characterizing the inequality seems difficult and, to our knowledge, is not in the literature. Let us rewrite Eq. (1) as

$$\begin{aligned} H(s,t)=F_{go,stop}(s,t)= C_{go,stop}(F_{go}(s),F_{stop}(t)), \end{aligned}$$

with $C_{go,stop}$ denoting a *copula*, that is a function that specifies how a bivariate (in general, multivariate) distribution is related to its one-dimensional marginal distributions, here $F_{go}(s)$ and $F_{stop}(t)$. A copula allows one to assess stochastic dependency between the two random variables $T_{go}$ and $T_{stop}$ separately from the choice of the marginal distributions. Thus, copulas are a natural tool to investigate which combination of stochastic dependency and marginal distributions will lead to a (non-)violation of Ineq. (11).

A formal definition of a copula for 2 variables is as follows:

### Definition 2

A 2-*dimensional copula *is a 2-dimensional distribution function *C* on the unit square $[0,1]^2$, whose univariate marginal distributions are uniformly distributed on [0, 1]:

$$\begin{aligned} C: [0,1] \times [0,1] \rightarrow [0,1] \end{aligned}$$

such that for any $u,v \in [0,1]$,

$$\begin{aligned} (u,v) \mapsto C(u,v) \in [0,1]. \end{aligned}$$

For more details on copula theory see the Appendix. A key result (see Appendix B), adapted to our context, is the following:

### Proposition 1

Let

$$\begin{aligned} H(s,t)= & {} P[T_{go}\le s, T_{stop}\le t]\\= & {} C_{go,stop}(F_{go}(s),F_{stop}(t)); \end{aligned}$$

the 2-dimensional copula *C* is determined uniquely assuming continuous marginal distributions. Moreover, with $F_{go}^{-1}$ and $F_{stop}^{-1}$ the inverse functions of $F_{go}$ and $F_{stop}$,

$$\begin{aligned} C_{go,stop}(u,v) = H(F_{go}^{-1}(u),F_{stop}^{-1}(v) ) \end{aligned}$$

for any $$(u,v) \in [0,1]^2$$.

This proposition shows that a copula *C* allows one to assess the stochastic dependency separately from the marginals. As a simple example, letting $u=F_{stop}(t)$ and $v=F_{go}(s)$, copula

$$\begin{aligned} C_{I\!N\!D}(u,v) \equiv u\,v \end{aligned}$$

defines stochastically independent race models.

A more complex case is the following:

### Example 1

(Bivariate Gaussian copula)

With $\Phi$ denoting the univariate standard normal distribution function and $\Phi _2(\cdot ,\cdot \,;\rho )$ the bivariate standard normal distribution with correlation $\rho$, the Gaussian copula is defined as

$$\begin{aligned} C_{Gauss}(u,v) =\Phi _2(\Phi ^{-1}(u),\Phi ^{-1}(v);\rho ). \end{aligned}$$

For $$H(s,t)= C_{Gauss}(F_{go}(s), F_{stop}(t))$$,

12 $$\begin{aligned} C(u,v)= & {} \int _{-\infty }^{\Phi ^{-1}(F_{go}(s))}\int _{-\infty }^{\Phi ^{-1}(F_{stop}(t))} \frac{1}{2\pi \sqrt{1-\rho ^2}} \nonumber \\{} & {} \,\exp \left( -\frac{s'^2-2\rho s' t' +t'^2}{2(1-\rho ^2)}\right) ds' \,dt', \end{aligned}$$

where the expression under the integrals is the bivariate standard-normal density with correlation $\rho$ defining the dependency separately from the marginals, and $F_{go}^{-1}$ and $F_{stop}^{-1}$ are the inverse functions of the marginals.

Because of the generality of the copula definition, the class of race models based on copulas obviously encompasses all race models with specified marginal distributions.

Since we are primarily interested in determining the stop signal distribution, we focus on Eq. 7:

$$\begin{aligned} F_{stop|go}(s-t_d|s)= 1-\frac{f_{sr}(s ;t_d)}{f_{go}(s)}\,p_r(t_d), \end{aligned}$$

Choosing the independence copula leads to the IND model without conditioning on $\{T_{go}=s\}$:

13 $$\begin{aligned} F_{stop} (s-t_d)&= 1-\frac{f_{sr}(s ;t_d)}{f_{go}(s)}\,p_r(t_d), \end{aligned}$$

as already derived in Colonius (1990a). Because all elements on the right-hand side of Eq. 13 are observable, this could provide an estimate for the stop signal distribution; however, simulation studies revealed that gaining reliable estimates requires unrealistically large numbers of observations (Band et al., 2003; Matzke et al., 2013).

For the general, non-independent case one may pick some copula *C*(*u*, *v*) and define random variables $U\equiv F_{go}(T_{go})$ and $V\equiv F_{stop}(T_{stop})$ uniformly distributed on [0, 1]. Letting $u=F_{go}(s)$ and $v=F_{stop}(t)$, from Lemma 1 in Appendix B,

14 $$\begin{aligned} \frac{\partial C(u,v)}{\partial u}&=\frac{\partial H(F_{go}^{-1}(u),F_{stop}^{-1}(v))}{\partial u}\nonumber \\&=\Pr [V\le v\,|\, U=u]\nonumber \\&=\! \Pr [ F_{stop}(T_{stop}) \!\le \! F_{stop}(t) \,|\,F_{go}(T_{go})\!=\!F_{go}(s)]\nonumber \\&=\Pr [ F_{stop}^{-1}\left[ F_{stop}(T_{stop})\right] \nonumber \\&\le F_{stop}^{-1}\left[ F_{stop}(t)\right] \,|\,F_{go}^{-1}\left[ F_{go}(T_{go})\right] \nonumber \\&=F_{go}^{-1}\left[ F_{go}(s)\right] ]\nonumber \\&=\Pr [T_{stop}\le t \,|\, T_{go}=s]. \end{aligned}$$

Gaining information about the (marginal) distribution of $T_{stop}$, this could be obtained by “integrating out” $T_{go}$:

15 $$\begin{aligned} F_{stop}(t-t_d)&= \int _{0}^{\infty } \Pr [T_{stop}+t_d\le t \,|\, T_{go}=s] \nonumber \\&\,f_{go}(s) \, ds, \;\; \text {for }t>t_d. \end{aligned}$$

Equation 7 reveals that all we can hope to obtain from data is information about the conditional distribution $\Pr [T_{stop}+t_d\le t \,|\, T_{go}=s]$ at points (*s*, *s*), so the conditional distribution under the integral is not available in full. This lack of information requires the model builder to settle for some copula. The following example illustrates this approach. Choosing a copula will be discussed in a subsequent section.

### Example 2

(Farlie-Gumbel-Morgenstern copula (FGM)) The FGM copula is defined as

16 $$\begin{aligned} C_{FGM}(u,v)&= u \, v [1 + \delta (1-u)\,(1-v)] \end{aligned}$$

with parameter $\delta \in [-1,1]$. This copula defines a stochastically dependent *semi-parametric* race model with bivariate distribution function

17 $$\begin{aligned} H_{FGM}(s,t)= & {} C_{FGM}(F_{go}(s),F_{stop}(t)) \nonumber \\= & {} F_{stop}(t) F_{go}(s) [1+\delta (1-F_{stop}(t))\nonumber \\{} & {} (1-F_{go}(s))], \end{aligned}$$

with parameter $\delta$ determining the strength of dependence between $T_{go}$ and $T_{stop}$. Setting $\delta =0$ corresponds to the independent race model, negative and positive values of $\delta$ to negative or positive dependent models, respectively. It is known that the FGM copula only allows for moderate levels of dependence (e.g. Kendall’s tau, $\tau \in [-2/9, 2/9]$).^2^

In order to obtain information about the processing time $T_{stop}$ via Eq. 7, we follow Eq. 14 and take the partial derivative of $C_{FGM}(u,v)$ with respect to *u*,

18 $$\begin{aligned} \frac{\partial C(u,v)}{\partial u}&=\Pr [V\le v\,|\, U=u]\nonumber \\&= v + \delta (2u-1)v(v-1) \end{aligned}$$

From Eq. 7 and inserting the distribution functions, we have

19 $$\begin{aligned}{} & {} F_{stop} (s-t_d\,|\, T_{go}=s) \nonumber \\= & {} F_{stop} (s-t_d) + \delta (2 F_{go}(s)-1) F_{stop} (s-t_d)\nonumber \\{} & {} ( F_{stop} (s-t_d) -1). \end{aligned}$$

The left-hand side, as well as $F_{go}(s)$, are in principle estimable so that Eq. 19, as a quadratic equation, can be numerically solved for both $F_{stop}$ and parameter $\delta$.

## The Copula Version of the Inequality

From Lemma 1 in Appendix B,

$$\begin{aligned} F_{stop|go}(t|s)&=\frac{\partial C_{go,stop}(F_{go}(s),F_{stop}(t))}{\partial F_{go}(s)}\\&=C_{stop|go}(F_{stop}(t)\,|\,F_{go}(s)). \end{aligned}$$

Then, for $s>t_d>0$, Eq. 5 becomes

20 $$\begin{aligned} \Pr [T_{go}\le & {} s, \, T_{go}<T_{stop}+t_d] \nonumber \\= & {} F_{go}(t_d) + \int _{t_d}^s [1-F_{stop|go}(s'-t_d|s')] \,\nonumber \\{} & {} f_{go}(s')\, \textrm{d}s' \nonumber \\= & {} F_{go}(s) -\int _{t_d}^s F_{stop|go}(s'-t_d|s') \, f_{go}(s')\, \textrm{d}s' \nonumber \\= & {} F_{go}(s) - \int _{t_d}^s C_{stop|go}(F_{stop}(s'-t_d) \,|\, F_{go}(s'))\,\nonumber \\{} & {} f_{go}(s')\,\textrm{d}s'. \end{aligned}$$

Let $s\rightarrow \infty$ to get:

$$\begin{aligned} \Pr [T_{go}<T_{stop}+t_d] = 1&- \int _{t_d}^\infty C_{stop|go}(F_{stop}(s' \\&-t_d) | F_{go}(s'))\, f_{go}(s')\,\textrm{d}s'. \end{aligned}$$

For $s>t_d$, Inequality (11) is the same as

$$\begin{aligned} \Pr [T_{go}\le s]{} & {} \Pr [T_{go}<T_{stop}+t_d] \le \\{} & {} \Pr [T_{go}\le s,T_{go}<T_{stop}+t_d], \end{aligned}$$

or,

$$\begin{aligned}{} & {} F_{go}(s)\Bigl [\!1\!-\! \int _{t_d}^\infty \!\!\!\!\!\! C_{stop|go}(F_{stop}(s'\!-\!t_d) | F_{go}(s'))\, f_{go}(s')\,\textrm{d}s' \!\Bigr ] \\\le & {} F_{go}(s)\! -\!\! \int _{t_d}^s\!\!\! C_{stop|go}(F_{stop}(s'\!-\!t_d) | F_{go}(s')) f_{go}(s')\,\textrm{d}s' \end{aligned}$$

or,

$$\begin{aligned}{} & {} F_{go}(s) \cdot \int _{t_d}^\infty \!\!\!\! C_{stop|go}(F_{stop}(s'-t_d) | F_{go}(s'))\, f_{go}(s')\,\textrm{d}s'\\\ge & {} \int _{t_d}^s\!\!\! C_{stop|go}(F_{stop}(s'\!\!-t_d) |\! F_{go}(s'))\, f_{go}(s')\,\textrm{d}s'. \end{aligned}$$

The final equation can be checked for different copulas $C_{go,stop}$ and parametric families for $F_{go}$ and $F_{stop}$. For $0<s\le t_d$, the inequality

$$\begin{aligned} \Pr [T_{go}&\le s] \Pr [T_{go}<T_{stop}+t_d] \\&\le \Pr [T_{go}\le s,T_{go}<T_{stop}+t_d] \end{aligned}$$

becomes

$$\begin{aligned} F_{go}(s) \Pr [T_{go}<T_{stop}+t_d] \le F_{go}(s), \end{aligned}$$

which is always satisfied.

### Sufficient Conditions for the Inequality

Several sufficient conditions for Inequality (11) to hold can be stated.

#### Proposition 2

Let *X* and *Y* be random variables with a joint density and conditional (cumulative) distribution function $F_{Y|X}(y|x)$. If $g(z)=1-F_{Y|X}(z|z)$ is decreasing in *z*, then

$$\begin{aligned} \Pr [X\le x]\le \Pr [X\le x\,|\, X\le Y] \end{aligned}$$

for all *x*.

(Note: “decreasing” is defined as non-increasing). The proof is in Appendix A. Replacing *X* by $T_{go}$ and *Y* by $T_{stop}+t_d$, the sufficient condition for Eq. 11 to hold for all *t* is that

$$\begin{aligned}{} & {} \Pr [T_{stop}+t_d > t \,|\, T_{go}=t]\nonumber \\= & {} 1\!-\!F_{stop|go}(t\!-\!t_d|t) \!=\! 1\!-\!C_{stop|go}(F_{stop}(t \!-\!t_d)|F_{go}(t)) \end{aligned}$$

is decreasing in $t>t_d$. Note that this condition is satisfied if $T_{go}$ is independent of $T_{stop}$.

#### Remark 1

From Eq. 7, the above implies that

$$\begin{aligned} \Pr [T_{stop}+t_d > t \,|\, T_{go}=t]&= \frac{f_{sr}(t; t_d)}{f_{go}(t)} \,p_r(t_d) \end{aligned}$$

is decreasing in *t*. Then, reversing the ratio

$$\begin{aligned} \frac{f_{go}(t')}{f_{sr}(t'; t_d)} \le \frac{f_{go}(t)}{f_{sr}(t; t_d)} \end{aligned}$$

holds for any $t' <t$, i.e. the ratio of densities has the *monotone likelihood property*. This is consistent with the intuition that, when observing a large value of *t*, it is more likely that it was drawn from distribution $f_{go}$ rather than $f_{sr}$.

#### Remark 2

From the proof of Proposition 2 (Appendix A) it is easily shown that the following holds as well: If $g(z)=1-F_{Y|X}(z|z)$ is *increasing*in *z*, then the inequality reverses:

$$\begin{aligned} \Pr [X\le x]\ge \Pr [X\le x\,|\, X\le Y] \end{aligned}$$

for all *x*.

A condition slightly stronger than assuming $g(z)=1-F_{Y|X}(z|z)$ is decreasing in *z* (Proposition 2) is given by the following:

#### Definition 3

*Stochastic decreasing negative dependence for* *Y* *given* *X* *(SDND)* means that $\Pr (Y>y|X=x)=1-F_Y(y|x)$ is decreasing in *x* for all *y*. If $C_{X,Y}$ is the copula of $F_{X,Y}$, then the condition is the same as $1-C_{Y|X}(v|u)$ decreasing in $u\in (0,1)$ for all $v\in (0,1)$ or $C_{Y|X}(v|u)$ increasing in $u\in (0,1)$ for all $v\in (0,1)$.

To show that SDND is indeed stronger, take $z_1 <z_2$. Then, by SDND, $\Pr (Y>z_1|X=z_1) \ge \Pr (Y>z_1|X=z_2)$; by monotonicity of the survival, $\Pr (Y>z_1|X=z_2)\ge \Pr (Y>z_2|X=z_2)$ showing that *g*(*z*) is decreasing.

If $T_{stop}$ is stochastically decreasing in $T_{go}$ in the sense of Definition 3, then Proposition 2 holds with $X=T_{go}$ and $Y=T_{stop}+t_d$. This generalizes the result of Colonius and Diederich (2018) with perfect negative dependence. However Proposition 2 with $X=T_{go}$ and $Y=T_{stop}+t_d$ can also hold with positive dependence of these two variables, depending on the relative *tail heaviness* of $F_{go}$ and $F_{stop}$.

The following example illustrates Proposition 2 and also allows one to find parameter settings violating the inequality.

#### Example 3

Let $(X,Y)^\prime$ be a bivariate normal random vector:

21 $$\begin{aligned} \begin{pmatrix}X \\ Y\end{pmatrix} \sim N\left( \begin{pmatrix}\mu _X\\ \mu _Y\end{pmatrix},\begin{pmatrix}\sigma _X^2 &{} \sigma _{XY}\\ \sigma _{XY}&{} \sigma _Y^2 \end{pmatrix}\right) . \end{aligned}$$

Then, for the conditional distribution,

$$\begin{aligned}{}[Y\,|\,X=x]\sim N(\mu _Y+\sigma _{XY}(x-\mu _X)/\sigma _X^2, \;\sigma _Y^2-\sigma _{XY}^2/\sigma _X^2) \end{aligned}$$

and, with $\Phi$ denoting the standard normal distribution function,

22 $$\begin{aligned} 1-F_{Y|X}(z|z)= & {} 1-\Phi \left( \frac{z-\mu _Y-\sigma _{XY}(z-\mu _X)/\sigma _X^2}{(\sigma _Y^2-\sigma _{XY}^2/\sigma _X^2)^{1/2}}\right) \\= & {} 1-\Phi \left( \frac{z(1-\sigma _{XY}/\sigma _X^2)-\mu _Y+\sigma _{XY}\mu _X/\sigma _X^2}{(\sigma _Y^2-\sigma _{XY}^2/\sigma _X^2)^{1/2}}\right) . \nonumber \end{aligned}$$

This is always decreasing in *z* if $1-\sigma _{XY}/\sigma _X^2 \ge 0$.

#### Remark 3

One can have $1-\sigma _{XY}/\sigma _X^2 =1- \rho _{XY} \,\sigma _Y/\sigma _X <0$ for $\rho _{XY}$ positive and $\sigma _Y/\sigma _X$ sufficiently large. An extreme case is as follows: let $\rho _{XY}=1, \mu _X=\mu _Y=0, \sigma _X^2=1$, and $Y=aX$ with $a>1$. Then $\sigma _Y=a \sigma _X= a$ and $1-\sigma _{XY}\sigma _Y/\sigma _X=1-a<0$. The event $\{X \le Y\}$ corresponds to $\{X \ge 0\}$ and

23 $$\begin{aligned} \Pr (X\le x\,|\,X\ge 0)= {\left\{ \begin{array}{ll} 0 &{}\text {if} x<0\\ 2 \Phi (x)-1 &{}\text {if} x\ge 0 \end{array}\right. } \quad \le \Phi (x) =\Pr (X\le x) \end{aligned}$$

violating the inequality for all *x*.

The next example illustrates the effect of varying SSD on the strength of violations of Inequality (11):

#### Example 4

(Ex-Gaussian marginals) Assume go and stop processing times follow an ex-Gaussian distribution^3^; thus, $T_{go}=N_{go} + E_{go}$ and $T_{stop}=N_{stop} + E_{stop}$ with $E_{go}, E_{stop}$ exponentially distributed and $N_{go},N_{stop}$ normally distributed random variables; moreover, stochastic independence is assumed throughout except for the pair $\{N_{go}, N_{stop}\}$ which has a bivariate normal distribution with correlation $\rho$.

**Fig. 1:**
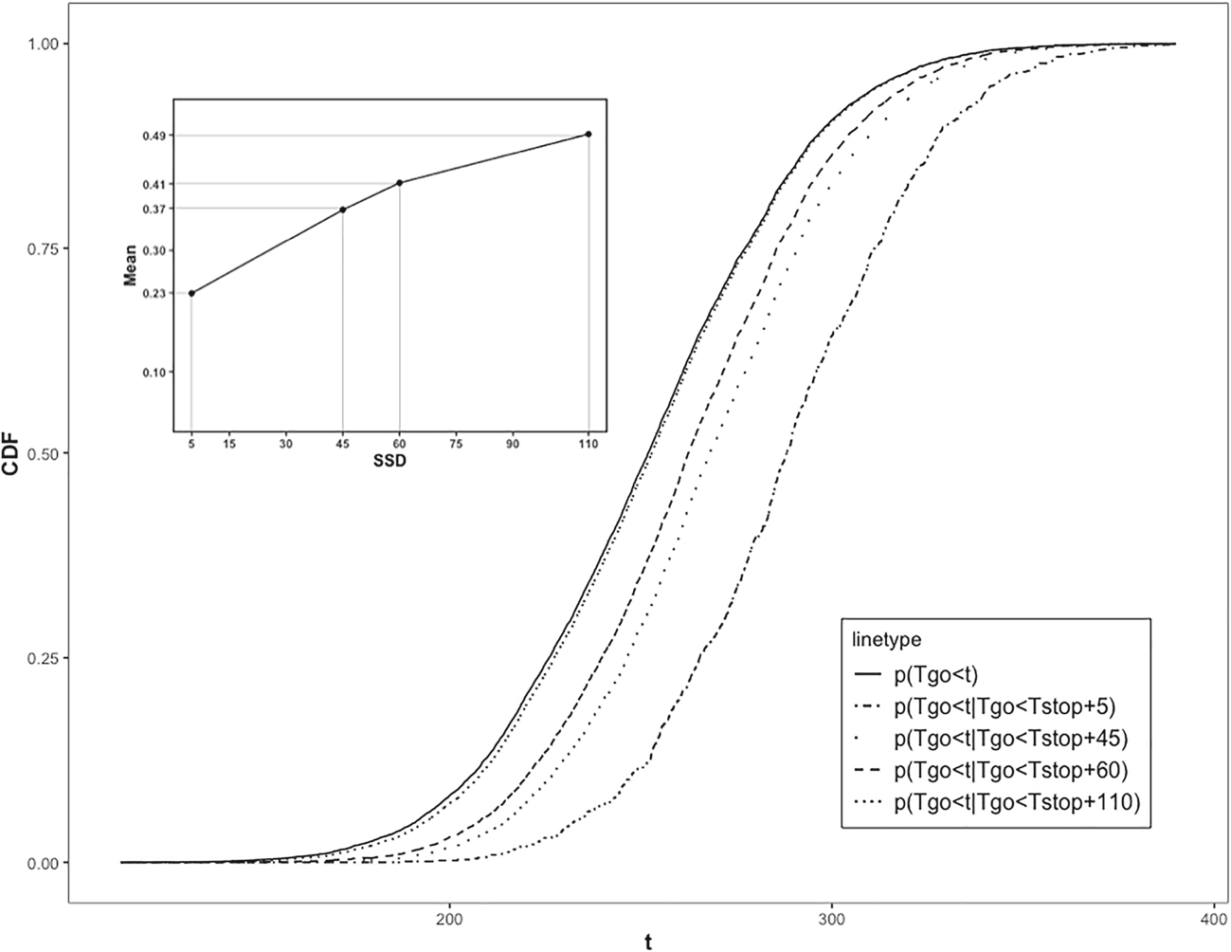
Violation of Inequality (11) as a function of stop signal delay. Go distribution ($F_{go}$) and signal-respond distributions ($F_{sr}$) from a correlated ($\rho =.9$) ex-Gaussian race model for SSD values 5, 45, 60, 110 [ms] and parameter values: $\mu _{go}=252, \mu _{stop}=204, \sigma _{go}=37, \sigma _{stop}=60$ [ms], and $\lambda =0.8$. Corresponding means are depicted in the upper-left inserted figure

As depicted in Fig. 1, the signal-respond distribution functions are ordered according to SSD values, with violation of Inequality (10) decreasing with increasing SSD.

A numerical evaluation by double integration is based on the following

$$\begin{aligned} (N_{go},N_{stop}) \sim N_2\left( (\mu _1,\mu _2),\begin{pmatrix} \sigma _1^2 &{} \rho \sigma _1\sigma _2 \\ \rho \sigma _1\sigma _2 &{} \sigma _2^2) \end{pmatrix}\right) \end{aligned}$$

truncated below at (0, 0), and

$$E_{go}\!\sim \!\mathrm{Exponential(rate=}\lambda )$$, $$E_{stop}\!\sim \!\mathrm{Exponential(rate=}\lambda )$$, with $$(N_{go},N_{stop}) \perp E_{go} \perp E_{stop}$$.

Let $Y=N_{go}$, $Z=N_{go}-N_{stop}$, so that $\textrm{E}\,(Z)=\mu _1-\mu _2$, $\textrm{Var}\,(Z)=\omega ^2=\sigma _1^2+\sigma _2^2-2\rho \sigma _1\sigma _2$, $\textrm{Cov}\,(Y,Z)= \sigma _1^2-\rho \sigma _1\sigma _2$, $\rho ^*=\textrm{Cor}\,(Y,Z)= (\sigma _1^2-\rho \sigma _1\sigma _2)/(\sigma _1\omega )$. Then

$$\begin{aligned} \Pr (T_{go}<t)= & {} \Pr (N_{go}+E_{go}<t) \\= & {} \int _0^\infty \Pr (N_{go}<t -e_1)\lambda e^{-\lambda e_1} \textrm{d}e_1 \\= & {} \int _0^\infty \Phi ((t\!-\!e_1\!-\!\mu _1) /\sigma _1) \lambda e^{-\lambda e_1} \textrm{d}e_1. \\ \end{aligned}$$

$$\begin{aligned}{} & {} \Pr (T_{go}\!\!<T_{stop}+t_d)\\= & {} \! \Pr (Z\!<\! -E_{go}\!+\!E_{stop}\!+t_d) \\= & {} \int _0^\infty \int _0^\infty \Phi ( (e_2-e_1 +t_d -(\mu _1-\mu _2))/\omega )\nonumber \\{} & {} \lambda ^2 e^{-\lambda e_1-\lambda e_2} \textrm{d}e_1 \textrm{d}e_2. \\ \end{aligned}$$

$$\begin{aligned}{} & {} \Pr (T_{go}<t,T_{go}<T_{stop}+t_d)\\= & {} \Pr (Y<-E_{go}+t,Z< -E_{go}+E_{stop}+t_d) \\= & {} \int _0^\infty \int _0^\infty \Phi _2\Bigl ({(t-e_1-\mu _1)\over \sigma _1},\nonumber \\{} & {} {(e_2-e_1+t_d-(\mu _1-\mu _2))\over \omega }; \rho ^*\Bigr ) \lambda ^2 e^{-\lambda (e_1+e_2)} \textrm{d}e_1 \textrm{d}e_2 \end{aligned}$$

where $\Phi _2(;\theta )$ is the bivariate normal cdf with zero means, unit variances and correlation $\theta$.

The next example illustrates Proposition 2 and Definition 3 using exponential marginals.

#### Example 5

(Gumbel’s bivariate exponential distribution)

24 $$\begin{aligned} H_{\theta }(x,y)&= {\left\{ \begin{array}{ll} 1-e^{-x}-e^{-y}\!+\!e^{-(x+y+\theta xy)}, &{}\!\!\! \text {if}\,\, x\!\ge \! 0,y\ge 0\\ 0, &{} \text {otherwise} \end{array}\right. } \end{aligned}$$

where $\theta$ is a parameter in [0, 1]. Then the margins are obviously exponential: $F(x)=1-e^{-x}$ and $G(y)=1-e^{-y}$ with inverses $F^{-1}(u)=-\ln (1-u)$ and $G^{-1}(v)=-\ln (1-v)$. Inserting $F^{-1}(u)$ and $G^{-1}(v)$ for *x* and *y*, respectively, in Eq. 24 yields its copula

$$\begin{aligned}{} & {} H_{\theta } (F^{-1}(u),G^{-1}(v))\nonumber \\= & {} u + v -1 +(1-u)(1-v)e^{-\theta \ln (1-u)\ln (1-v)}\nonumber \\= & {} C_{\theta }(u,v) . \end{aligned}$$

Then,

25 $$\begin{aligned} H_{\theta }(y|x)&= 1-e^{-y(1+\theta x)}(1+\theta y). \end{aligned}$$

Replacing (*X*, *Y*) with random variables $(T_{go}, T_{stop})$

$$\begin{aligned} H_{\theta }(s,t;t_d)&= C(F_{go}(s),F_{stop}(t-t_d))\\&= 1-e^{-s}-e^{-(t-t_d)}+e^{-(s+t-t_d+\theta s (t-t_d))}, \end{aligned}$$

with $s\ge 0, t-t_d\ge 0$. From Eq. 25, the conditional df is

$$\begin{aligned} H_{\theta }(t|s;t_d)&= \Pr (T_{stop}\le t-t_d\,|\,T_{go}=s)\\&=1-e^{-(t-t_d)(1+\theta s)}(1+\theta (t-t_d)) \end{aligned}$$

with $s\ge 0, t-t_d\ge 0$. Obviously, $1-H_{\theta }(t|s;t_d)$ is decreasing in *t*, so by Proposition 2, Inequality (11) is satisfied for all $t\ge t_d$. Note that this already follows via Definition 3 because Gumbel’s bivariate exponential distribution is known to have negative dependence.

The exponential marginals could be replaced by more realistic distributions like Weibull or log-normal. However, given that we only have a sufficient condition for Inequality (11) to hold, we cannot draw general conclusions about the effect of choosing arbitrary marginals on the violation.

## Choosing a Copula: The Role of Dependent Random Censoring

As noted above, due to the limited observability expressed in Eq. 7 the model builder faces a non-identifiability problem. Interestingly, it turns out that this problem is formally equivalent to a classic one studied in actuarial science, an area concerned with the determination of the *time of failure* of some entity, e.g. human or machine. We first show the equivalence and then sketch some recent developments in actuarial science and survival modeling relevant to the non-identifiability issue in stop signal modeling.

### Equivalence with Dependent Random Censoring

*Censoring* is a condition in which the failure time is only partially known. In the case of *right censoring* a data point is above a certain value but it is unknown by how much. For example, in medical studies, one is often interested in the survival time *T* of patients who will die of a certain disease. However, it often happens that patients remain alive at the end of the study, or leave the study before the end for various reasons, or die from another cause at some time point *X* referred to as observation time (or random censoring time). If $X<T$ the survival time is not observable. Thus, one can directly estimate only the following two functions:

$$\begin{aligned} G(x)&= \Pr (X\le x) \quad \text {and} \quad p_2(x) \\&=\Pr (\{X\le x\}\cap \{T<X\}), \quad (0\le x \le \infty ) \end{aligned}$$

by their empirical estimates. The following shows the formal equivalence with the dependent race model:

#### Remark 4

(Colonius and Diederich , 2023) We equate distribution *G*(*x*) with $F_{go}(s)$ and *F*(*t*) with $F_{stop}(t)$. Thus, $p_2(x)=\Pr (\{X\le x\}\cap \{T<X\})$ corresponds to $\Pr (\{T_{go}\le s \}\cap \{T_{stop}+t_d< T_{go}\})$. Since the latter is not observable, we use the following equality,

$$\begin{aligned}&\Pr (T_{go}\le s) - \Pr (\{T_{go}\le s \}\cap \{T_{stop}+t_d< T_{go}\})&\\&= \Pr (\{T_{go}\le s \}\cap \{ T_{go}< T_{stop}+t_d\})&\\&= \Pr (T_{go}\le s\,|\, T_{go}< T_{stop}+t_d)\, \Pr (T_{go}< T_{stop}+t_d)&\\&= F_{sr}(s\,|\,t_d)\, [1-p_r(t_d)], \end{aligned}$$

showing a one-to-one correspondence between the observable quantities in dependent censoring and the stop signal race model; note that we made use of the correspondence of $p_2(\infty )$ with $\Pr (T_{stop}+t_d < T_{go})\equiv p_r(t_d)$.

### Potential Consequences for Identifiability in Stop Signal Modeling

In most work on censoring it is assumed that the survival time *T* is stochastically independent of the censoring time *X*, but there are many instances in which this independence assumption is violated. Consider, for example, the case where the patient dies of another related disease, and such dependency should be taken into account in the model.^4^

With *F*(*t*) and *G*(*x*) the distribution functions of *T* and *X*, respectively, one assumes a copula *C*

$$\begin{aligned} C(F(t),G(x)) \end{aligned}$$

to specify the dependence between failure time and observation time. Modeling random dependent censoring has recently become an active area of actuarial science and statistics (Emura & Chen, 2018; Hsieh & Chen, 2020).

The formal equivalence to stop signal-dependent race models just established opens up a host of results potentially relevant to the copula approach suggested here. The approaches differ in whether they make parametric assumptions on the distributions of *T* and *X*, consider a completely known copula, or only a semi-parametric model; unsurprisingly, (non-)identifiability depends on the specifics of these assumptions. For example, if the copula is known, the non-observable failure time distribution can be determined uniquely given the observable data *G*(*x*) and $p_2(x)$ (Wang et al., 2012). Translated to the stop signal model, this means that $F_{stop}(t)$ is uniquely determined in the context-independent race model with a specified copula. There is a caveat concerning identifiability of the dependence parameter: a well-known result implies that the numerical value of the dependence parameter, e.g. of $\delta$ in the case of the FGM model, is not in general identifiable (Betensky, 2000) but, as noted in (p.457 Titman 2014) it will often be possible to estimate both the dependence parameter and the parameters of the margins simultaneously.

Most recently, Czado and Van Keilegom (2023) present sufficient conditions on the margins and the partial derivatives of copula *C*(*F*(*t*), *G*(*x*)) to identify the joint distribution of (*T*, *X*) including the association parameter of the copula. The margins and the copula remain basically unspecified except for assuming that they belong to some parametric family. Identifiability means here that the parameters uniquely determine the densities of the observable random variables. Given the central role of the copula partial derivative in our approach (Eq. 14), this seems to be a good starting point for further investigation of copula-based race models for the stop signal paradigm.

## Summary and Discussion

The race model for stop signal processing is based on two important assumptions of independence between the go and stop process: stochastic independence and context independence. Recent empirical evidence violating predictions of this model has been interpreted as a failure of context independence (e.g., Bissett et al. 2021). In addition, design issues in a large-scale study of brain development and child health (ABCD study) (Casey et al., 2018), have raised doubts about the validity of context independence and prompted the development of some alternative versions of the race model dispensing with the assumption (Bissett et al., 2021; Weigard et al., 2023). The ubiquity of context independence violations is currently under discussion. A recent study by Doekemeijer et al. (2023) did not find evidence of violations suggesting that it may depend on the complexity of the stopping task. In a similar vein, both theoretical and empirical evidence for context independence violations exists for variations on the stop signal paradigm. A case in point is *stimulus selective stopping* (e.g., Bissett and Logan 2014), where two different signals can be presented on a trial, and participants must stop if one of them occurs (stop signal), but not if the other occurs (ignore signal). It has been hypothesized that in these tasks, the decision to stop or not will share limited processing capacity with the go task. When the decision is difficult, the go and stop task will have to share capacity for a longer period, resulting in longer RTs on stop signal trials (Verbruggen & Logan, 2015).

In sum, further research about the status of context independence seems necessary (see also below).

The important points that we demonstrate here are, first, that assuming stochastic dependency between go and stop processing can also account for observed violations of the independent (IND) model. In particular, sufficient conditions for a failure of the critical distribution inequality are presented, implying a reversal of the predicted ordering of the means for go and signal respond processing. This is all the more intriguing as it seems unlikely that one can pinpoint which type of violation of independence– context or stochastic– is causing the failure of the IND model without adding assumptions that would themselves need further justification.

Second, in order to introduce stochastic dependency we expound on the critical role of the concept of copulas, a rapidly developing area of statistics (e.g., Joe 2014). After providing the general feasibility of deriving race models from copulas, several detailed examples illustrated the approach. Applying a copula to our paradigm is not straightforward, however, because due to the non-observability of stop signal processing time, one of its marginals has to be derived rather than simply estimated from data. Fortunately, we can show that this problem is formally equivalent to a well known one posed in random dependent censoring, an active area of actuarial science (e.g., Crowder 2012). We report on a number of recent results on (non-) identifiability in this field, depending on specific assumptions, that suggest to be of relevance for stop signal modeling.

Given our results, we feel that discussing the distinction between context and stochastic independence has mostly been too superficial, if not lacking entirely, in the stop signal literature up to now; but see Verbruggen and Logan (2015). Whenever there is empirical evidence–whether behavioral or neural– for shortcomings of the IND model, context independence is typically pointed at as causing the problem (e.g., Schall et al. , 2017; Bissett and Poldrack , 2022). However, there may actually be good reasons not to drop it at all if one wants to retain the basic idea of a race model.

The argument comes from modeling a related paradigm, the*redundant signals detection task* for simple reaction time^5^ (e.g., Miller 1982). In an elaborate analysis of the notion of “interactive race”, (Miller, 2016) shows, with a simple formal argument, that dropping context independence makes race models unfalsifiable, that is, any observed redundant-signals reaction time distribution is explainable perfectly within a context-dependent race if no further constraints are added. In other words, context independence is an elemental feature of race models. Given that observability in the stop signal task is even more limited than in the redundant signals task, the conclusion also applies to this paradigm. It should be noted, however, that explicit cognitive processing models using parametric assumptions do not necessarily require context independence and are still empirically testable.^6^

Turning to stochastic dependency, it is important to realize that the choice of a certain copula does not automatically imply the specific type of dependency. For example, in a semi-parametric copula like the FGM copula (see Example 2), it depends on the sign of the association parameter whether positive or negative dependence occurs. Moreover, the parameter value in the copula does not accurately reflect the strength of the dependency; taking again the FGM copula, the extremal values of its parameter $\delta$, $-1$ and $+1$, do not correspond to extremal correlation values but, e.g., $\tau =2\delta /9$ for Kendall’s tau.

What can be said about the sign of dependency between go signal and stop signal processing time? In our ex-Gaussian example (Example 4) high positive correlation was required to elicit violation of the distribution order inequality, $F_{go}(t) \le F_{sr}(t\,|\,t_d)$. On the other hand, findings from the neurophysiology of saccadic countermanding have shown that the neural correlates of go and stop processes consist of networks of mutually interacting gaze-shifting and gaze-holding neurons. This has been interpreted as creating a paradox between neural and behavioral modeling (Boucher et al., 2007; Schall et al., 2017): How can interacting circuits of mutually inhibitory neurons instantiate stop and go processes with stochastically independent finishing times? In an effort to bring these findings in line, we developed a generalization of the IND race model that allows for perfect negative dependency between the processes^7^ Colonius and Diederich (2018). This model, however, just like the IND model, does not predict violations of the distribution order inequality.

Thus, the final verdict about the “true” nature of dependency between going and stopping seems still standing out. There is a possibility that limiting the alternatives to just positive or negative dependence, or independence, is not appropriate. Schall et al. (2017) tried to resolve the above paradox by assuming that the go and stop processes are developing independently “most of the time” with a strong and quasi-instantaneous interaction at the end of processing. Generalizing this idea, Bissett et al. (2021) simulated a (preliminary) interactive model where the potency of the stop signal to inhibit the go process is varying across trials according to a Gaussian distribution. They found strong violations of the IND model only at short SSDs and could explain this by the different time spans available for a weak stop signal to affect the go process.^8^ Both these versions of an interactive race are claimed as relaxing the context independence assumption of the IND model. Because the models are not fully formalized, this is difficult to argue with. However, there may be ways to develop race models for these interactive processes drawing upon local dependency measures. It is well known that a single signed measure like Pearson’s correlation or Kendall’s tau cannot capture non-linear dependencies in variables where, for some regions of support, dependence is stronger (positive or negative) and for other regions it is weaker. A specific approach capturing such more complex dependency structures is called *local Gaussian correlation* (Tjostheim and Hufthammer , 2013; Tjostheim et al. , 2022) and this may deserve closer scrutiny.

## Data Availability

Not applicable

## Appendix A: Proof of Proposition 2

Assume $\Pr (X\le Y)>0$. Let $\overline{F}_{Y|X} = 1- F_{Y|X}$ and note that $h(z)=I_{(-\infty ,x)}(z)$ is decreasing where *I* denotes the indicator function. Fix *x* in the support of *X*. *X* is continuous with density $f_X$. Then

$$\begin{aligned} \Pr (X\le x\,|\,X\le Y)&=\frac{\Pr (X\le x, X\le Y)}{\Pr (X \le Y)} \\&= \frac{\int _{-\infty }^x \overline{F}_{Y|X}(z|z) f_X(z) \,dz}{\int _{-\infty }^{\infty } \overline{F}_{Y|X}(z|z) f_X(z) \,dz}\\&= \frac{\int _{-\infty }^{\infty }I_{(-\infty , x)}(z) \overline{F}_{Y|X}(z|z) f_X(z) \,dz}{\int _{-\infty }^{\infty } \overline{F}_{Y|X}(z|z) f_X(z) \,dz} \end{aligned}$$

and

$$\begin{aligned} \Pr (X \le x)= \int _{-\infty }^{\infty } I_{(-\infty ,x)}(z) f_X(z)\,dz. \end{aligned}$$

By assumption, $g(z)= \overline{F}_{Y|X}(z|z)$ is decreasing in *z*. The covariance of two decreasing functions is non-negative (if it exists, see e.g. Egozcue et al. , 2009). Since *g* and *h* are bounded functions,

$$\begin{aligned} \textrm{cov} (g(X),h(X)) \ge 0 \;\;\; \text {or} \;\;\; \text{ E }[g(X) \,h(X)] \!\ge \! \text{ E }[g(X)] \; \text{ E }[h(X)]. \end{aligned}$$

With the definitions of *g*, *h*,

$$\begin{aligned} \text{ E }[g(X) \,h(X)] =&\int _{-\infty }^{\infty }I_{(-\infty ,x)}(z)\,\overline{F}_{Y|X}(z|z)\,f_X(z)\,dz\\ \text{ E }[g(X)]=&\int _{-\infty }^{\infty } \overline{F}_{Y|X}(z|z)\,f_X(z)\,dz\\ \text{ E }[h(X)] =&\int _{-\infty }^{\infty } I_{(-\infty ,x)}(z)\,f_X(z)\,dz. \end{aligned}$$

The inequality $\Pr (X\le x, \,X\le Y)\ge \Pr (X \le Y) \,\Pr (X\le x)$ follows.

## Appendix B: Copula theory background

We only consider two-dimensional ($d=2$) copulas here; for the general case, we refer to Nelsen (2006); Joe (2014) and Durante and Sempi (2016). Proposition 2 follows directly from Sklar’s theorem, a simple version of which is as follows:

### Proposition 3

(a) For a bivariate distribution *H* with margins $H_1$ and $H_2$, the copula *C* associated with *H* exists with uniform on [0, 1] margins

B1 $$\begin{aligned} H(x_1,x_2) = C(H_1(x_1), H_2(x_2)) , \end{aligned}$$

for $$(x_1,x_2) \in \mathbb {R}^2$$.

(b) If *H* is a bivariate distribution function with continuous margins $H_1, H_2$ and quantile functions (inverses) $H_1^{-1}, H_2^{-1}$, then

B2 $$\begin{aligned} C(u_1,u_2) = H(H_1^{-1}(u_1), H_2^{-1}(u_2)), \end{aligned}$$

for $$(u_1,u_2) \in [0,1]^2$$.

The proof follows essentially from two properties:(i) If *F* is a univariate cdf and $Y\sim F$ ($\sim$ means “distributed as”), then $F(Y)\sim \textrm{U}(0,1)$; (ii) if *F* is a univariate cdf with inverse $F^{-1}$ and $U \sim \textrm{U}(0,1)$ , then $F^{-1}(U)\sim F$. Hence, if $(X_1,X_2)\sim H$, then $(H_1(X_1),H_2(X_2))\sim C$, and if $(U_1,U_2)\sim C$, then $(H_1^{-1}(U_1),H_2^{-1}(U_2))\sim H$.

We also need this property of conditional (cumulative) distribution functions (cdfs):

### Lemma 1

For random variables $X_1,X_2$ the conditional cdf is defined as

B3 $$\begin{aligned} H_{2|1}(x_2|x_1)&= \Pr (X_2 \le x_2\,|\, X_1=x_1) \nonumber \\&=\lim _{\epsilon \rightarrow 0^+} \frac{\Pr (x_1\le X_1<x_1+\epsilon ,X_2 \le x_2)}{\Pr (x_1\le X_1<x_1+\epsilon )} \nonumber \\&= \lim _{\epsilon \rightarrow 0^+} \frac{\Pr (x_1\le X_1<x_1+\epsilon ,X_2 \le x_2)}{\epsilon } \, \nonumber \\&\quad \lim _{\epsilon \rightarrow 0^+}\frac{\epsilon }{\Pr (x_1\le X_1<x_1 +\epsilon )}\nonumber \\&= \frac{\partial H_{12}(x_1,x_2)}{\partial x_1}/ h_1(x_1) \end{aligned}$$

For the special case of $(X_1,X_2)= (U_1,U_2)$ (that is, $U_i \sim \textrm{U}(0,1)$):

B4 $$\begin{aligned} H_{2|1}(x_2|x_1)&=C_{2|1}(u_2|u_1)\nonumber \\&= \frac{\partial C_{12}(u_1,u_2)}{\partial u_1}/1 \nonumber \\&= \Pr (U_2\le u_2\,|\, U_1=u_1). \end{aligned}$$

## Footnotes

1 *Context invariance* seems more fitting but we keep the familiar term *context independence* here.

2 FGM copula extensions with a slightly larger dependency range can be constructed but require additional parameters (see the copula literature in the references).

3 The Gaussian assumption permits positive probability for negative values of $N_{go}$ and $N_{stop}$, but they are negligible given the parameter values.

4 A slightly more general case is when there are several possible cases of failure. Thus, the object (machine, person, etc.) is simultaneously exposed to several, mutually exclusive and collectively exhaustive, causes of failure (*competing risks* situation). Suppose that only the time of failure and the identity of its cause are observable. A classic non-identifiability result is that under weak conditions it is always possible to generate the data as if the different risks act as stochastically independent random variables (Tsiatis, 1975). This result has found applications in non-parametric, parallel models of reaction time and choice (e.g., Marley and Colonius , 1992; Townsend 1976).

5 The participant’s task is to press a button as soon as a signal is detected, say, either a visual or an auditory stimulus, and both single-modality and bimodal stimuli are presented over different trials. In a simplified model version, let *V* and *A* be the time to process the visual and auditory stimulus, respectively, and to prepare and execute the response. The “race model” (Raab, 1962; Colonius, 1990b; Diederich, 1995; Diederich & Colonius, 1987) holds that the response in the bimodal condition is determined by the minimum time, $\min \{V,A\}$. In close correspondence with the stop signal race model, *context independence* here means that the distribution of *V* and *A* in the single stimulus conditions has to be equal to the margins of the bivariate distribution of (*V*, *A*) in the bimodal condition.

6 For example, in the racing-diffusion evidence-accumulation model (RDEX–ABCD) (see Weigard et al. , 2023; Tanis et al. , 2022), developed to describe the context independence violations in the ABCD study (Casey et al., 2018), to account for the effect of visual masking on evidence accumulation for go choices at short SSDs, the go process has an accumulation rate that changes with SSD up to an asymptotic level that matches go trials with no masking.

7 Assuming the *countermonotonicity* copula (of perfect negative dependence).

8 Bissett et al. (2021, p. 4–5):“When cross-trial variability in stop potency was instantiated at all SSDs, this resulted in large violations of context independence only at short SSDs, even though the same variability was present in longer-SSD trials. This is because when SSDs were long and inhibition was weak, the go process was already near completion when the weak inhibition began, so it had little opportunity to slow the go proces , and stop failure RTs were largely unaffected by this weak inhibition (Fig. 2b). However, when SSDs were short and inhibition was weak, the go process had just started when the weak inhibition began, and therefore the interaction between the weak stop process and the go process had more time to unfold, which resulted in more severe slowing of simulated stop-failure RTs and, in turn , severe simulated violations of context independence (Fig. 2c). When stop potency is allowed to vary, a prolonged dependency between the go and stop processes arises on weak-inhibition trials, particularly at short SSDs.”

## Code Availability

Available on request from the authors
